# Experimental Study on Compressive and Flexural Performance of Lightweight Cement-Based Composites Reinforced with Hybrid Short Fibers

**DOI:** 10.3390/ma16124457

**Published:** 2023-06-19

**Authors:** Cong-Thuat Dang, My Pham, Ngoc-Hieu Dinh

**Affiliations:** Faculty of Civil Engineering, The University of Danang—University of Science and Technology, 54 Nguyen Luong Bang, Danang 550000, Vietnam; phammy@dut.udn.vn

**Keywords:** fiber-reinforced cement-based composites, short fibers, compressive strength, flexural strength, tensile toughness

## Abstract

This paper aims to experimentally study the compressive and flexural characteristics of cement-based composites developed for fabricating thin, lightweight, and high-performance components of buildings. Expanded hollow glass particles with a 0.25–0.5 mm particle size were used as lightweight fillers. Hybrid fibers made of amorphous metallic (AM) and nylon fibers were used to reinforce the matrix with a total volume fraction of 1.5%. The primary test parameters included the expanded glass-to-binder (EG/B) ratio, the fiber volume content ratio, and the length of the nylon fibers in the hybrid system. The experimental results demonstrate that the EG/B ratio and the volume dosage of the nylon fibers exhibited insignificant effects on the compressive strength of the composites. Additionally, the utilization of nylon fibers with a longer length of 12 mm resulted in a slight compressive strength reduction of approximately 13% compared to that of the 6 mm nylon fibers. Further, the EG/G ratio exhibited an insignificant effect on the flexural behavior of lightweight cement-based composites in terms of their initial stiffness, strength, and ductility. Meanwhile, the increasing AM fiber volume fraction in the hybrid system from 0.25% to 0.5% and 1.0% improved flexural toughness by 42.8% and 57.2%, respectively. In addition, the nylon fiber length significantly affected the deformation capacity at the peak load and the residual strength in the post-peak stage.

## 1. Introduction

Nowadays, lightweight cement-based composites are used popularly in structural buildings to replace conventional normal-weight counterparts because they possess many outstanding advantages, such as low density, high thermal conductivity, and a high-temperature resistance capacity. In addition, the self-weight of the structure plays an essential factor in the design and construction of buildings. To reduce the self-weight of the structure, lightweight concrete with high performance is one of the critical issues in balancing the relationships among the strength, ductility, and structural self-weight of concrete materials. The practical need to produce high-performance, lightweight concrete has increased in recent years. In high-rise buildings, structural high-performance, and lightweight concrete can significantly reduce the self-weight load and, therefore, mitigate the effects of dynamic loads, such as strong winds or earthquakes. Li et al. [1] studied the shear behavior of beams made of steel fiber-reinforced expanded-shale lightweight concrete (SFRELC) by four-point bending tests. The experimental results indicate that the shear failure modes of SFRELC beams with stirrups were altered from brittle to ductile with an increasing fiber volume fraction. De Maio et al. [2] evaluated the behavior of reinforced concrete (RC) structures strengthened with an FRP-based system subjected to progressive damage by the numerical method and analyzed the experimental results. The numerical fracture model based on the cohesive crack approach incorporating an embedded truss model was used in the study to simulate the damage to RC structures under quasi-static loading conditions. The numerical results showed the beneficial effects of the FRP strengthening system on both the static and dynamic response in terms of the load-carrying capacity and the degradation of the natural vibration frequency. In another study by De Maio et al. [3], a numerical investigation of the reinforcing effect of nano-modified epoxy resin on the response of FRP-retrofitted RC components was conducted. The integrated model based on a cohesive crack approach incorporating bond–slip behavior was employed to carry out a failure analysis of retrofitted structures and verified by the previous experimental study. As a result, the reliability of the proposed model was proven by the good agreement between the analytical and experimental results.

**Figure 1:**
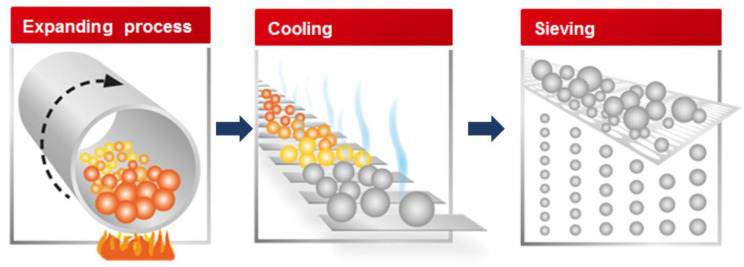
The processes of expanding, cooling, and sieving EG particles (reproduced from Sommariva and Weinberger [7], AIDIC/CET is acknowledged as source and the copyright is respected).

Lightweight cement-based composites with high performance are particularly essential for the rapid development of the prefabricated industry because the manufactured structural members are thin, tidy, and well utilized in a modular integrated structure, thereby reducing transportation, machining, and installation costs, etc. The most potential direction to produce lightweight cement-based composites is by using lightweight aggregates (LWA) combined with a cement-based matrix. Over the last several decades, many different types of LWAs have been investigated in various studies. Fundamentally, LWAs can be classified into artificial aggregates (e.g., expanded clay and shale) and natural aggregates (e.g., pumice and scoria). Gadea et al. [4] conducted an experimental study to produce lightweight mortar by using rigid polyurethane foam waste with a particle size of less than 4 mm. Several mortar grades were achieved by mixing cement with different amounts of polyurethane, aggregate, and water. The study pointed out that when the amount of polyurethane increased, the mortar density and mechanical properties were reduced. In contrast, its workability, permeability, and air content increased. Alduaij et al. [5] studied lightweight concrete in hot coastal regions using expanded clay as an LWA. The analyzed results showed that the lightweight concrete reached a compressive strength of 5.5–29 MPa when increasing the cement content from 250 to 350 kg/m^3^, while its densities were almost constant, around 1500 kg/m^3^. Recently, a new type of LWA, expanded glass (EG), was introduced as a substitute for conventional aggregates. EG is a product of industrial waste materials. It is very stable because its production occurs in a special kiln at 900 °C. Further, it is well-suited for LW mortar or concrete production processes due to its high strength, durability, pressure resistance, and environmental friendliness [6]. Figure 1 shows the processes of expanding, cooling, and sieving glass particles (Sommariva and Weinberger [7]). The raw materials react with each other, become viscous, and sinter, followed by the expansion of the agent and the formation of gas and pore structures at approximately 600 °C. After achieving optimum levels of the number of pores, cell size, and thickness in the temperature range of 800–900 °C, a cooling process is then initiated to lower the temperature to below 100 °C, followed by a sieving process for particle size classification. Carsana and Bertolini [8] used EG aggregate and silica fume to replace the fine fraction of aggregate and mineral addition to produce lightweight concrete with a density of less than 1800 kg/m^3^ and compressive strength of approximately 25 MPa. The results demonstrated that besides increasing the cohesiveness of fresh concrete, the low water-to-binder (W/B) ratio, and the presence of silica fume, the concrete was also effectively resistant to the intense penetration of sulfate and chloride ions. Rumsys et al. [9] performed an experimental study on the compressive strength and durability properties of lightweight concrete using fine EG and different types of micro-fillers. This study found that the durability of the investigated concrete using fine EG was sufficient. Therefore, the considered lightweight concrete mixtures can be applied to produce structural elements that require high durability. Lightweight EG particles were also studied recently by Adhikary et al. [6] to develop flowable ultra-lightweight concrete, which can be used as a sustainable material for energy-efficient buildings. The test results concluded that a concrete density of less than 800 kg/m^3^ could be achieved using a higher content of aerogel particles incorporating EG. In addition, fly ash and prefabricated plastic bubbles were found to be very beneficial for manufacturing LW and ultra-LW concretes.

Owing to the existence of LWAs inducing an increase in the porosity within cement-based matrices, the mechanical properties and ductility of lightweight concrete are usually poor compared to normal-weight concrete. Dispersed micro-fibers [10] have been incorporated in several studies to increase the mechanical performance of lightweight cement-based composites. Iqbal et al. [11] investigated the addition of micro-steel fiber in high-strength, lightweight, and self-compacting concrete. The results showed that the compressive strength decreased by about 12%, corresponding to a steel fiber content greater than or equal to 1%. Meanwhile, the splitting tensile and flexural strength increased by about 37% and 110%, respectively, when the steel fiber content increased from 0% to 1.25%. Li et al. [12] compared the effectiveness of polypropylene fibers (PPFs) and steel fibers (SFs) for fabricating highly ductile lightweight concrete. The results from this study pointed out that the optimal contents for the PPFs and SFs were 1.1% and 2.0%, respectively. Among the two fiber types, the SFs exhibited better performance than the PPFs in improving the initial and residual flexural strength, whereas the PPFs exhibited better performance in the post-peak stage.

Despite the previous literature, further research on lightweight cement-based composites with high performance still has much potential for development. It is still attracting a lot of attention today. The present research aimed to investigate the compressive and flexural properties of a newly developed type of high-performance, lightweight cement-based composites using short hybrid fibers. EG particles made from industrial refractory materials with a 0.25–0.5 mm particle size were used as lightweight fillers. A hybrid fiber type made up of short amorphous metallic (AM) and nylon fibers was employed to reinforce the cementitious matrix. The fibers in this study were utilized in the form of short types to ensure their workability and fiber distribution issues and their appropriateness for thin structures. The primary test parameters included the expanded glass-to-binder (EG/B) ratio, the fiber volume content ratio, and the length of nylon fibers in the hybrid system. The contribution of this study can provide useful insight into construction materials for fabricating thin, lightweight, and high-performance structural elements of buildings, such as slabs, structural walls, and coupling beams.

## 2. Materials and Experimental Layout

### 2.1. Material Properties

**Figure 2:**
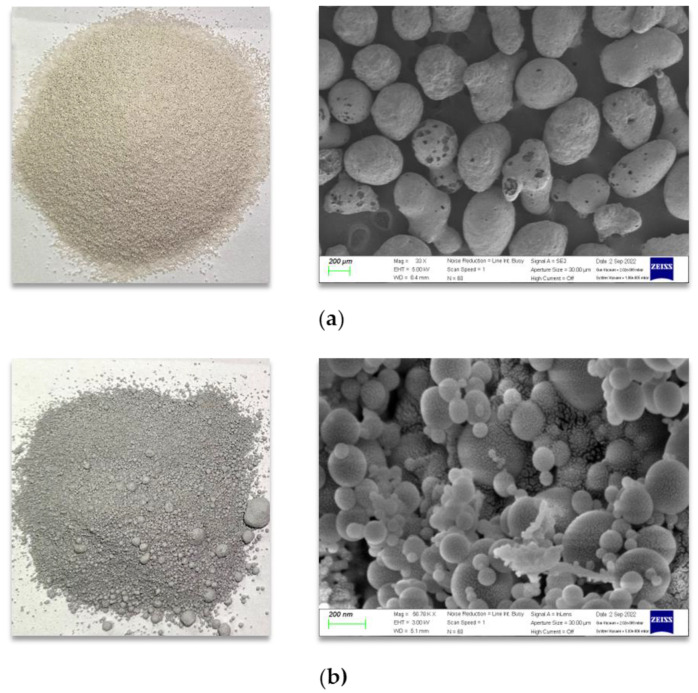
Morphologies of expanded glass particles and microsilica: (**a**) morphology of EG particles; (**b**) morphology of microsilica.

Fine EG particles made from industrial refractory materials with a grain size range of 0.25–0.5 mm were utilized as lightweight fillers. Table 1 summarizes the main physical and mechanical properties of the EG provided by the manufacturer. Figure 2a shows the morphology and scanning electron microscope (SEM) images of the EG.

**Figure 3:**
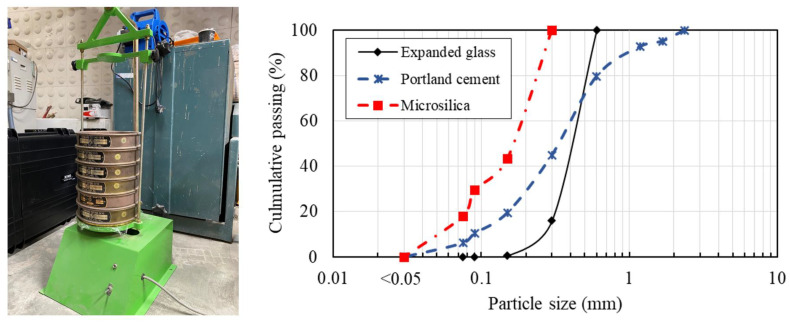
Particle size distributions of components for cement-based matrix.

Table 2 presents the developed lightweight cement-based mixtures. The main parameter of the mix proportions is the EG dosage. The mix ID of LCEG02 and LCEG03 used the EG/B ratios of 0.2 and 0.3, respectively. The water-to-cement ratio was kept at a constant of 0.35 for all mixtures. Microsilica (Figure 2b) was used as supplementary cementing material to improve the density of the matrix and the bond quality between the lightweight particles and the cement paste (Wang et al. [17], Rashad et al. [18]). Figure 3 presents the particle size distribution curves of the EG, microsilica, and Portland cement obtained from vibration tests in accordance with ASTM C33 [19], conducted using the sieve machine. The chemical composition, clinker composition minerals, and physical properties of the cement and microsilica are shown in Table 3. A commercial superplasticizer (SP) was used to achieve suitable workability of the mixture.

**Figure 4:**
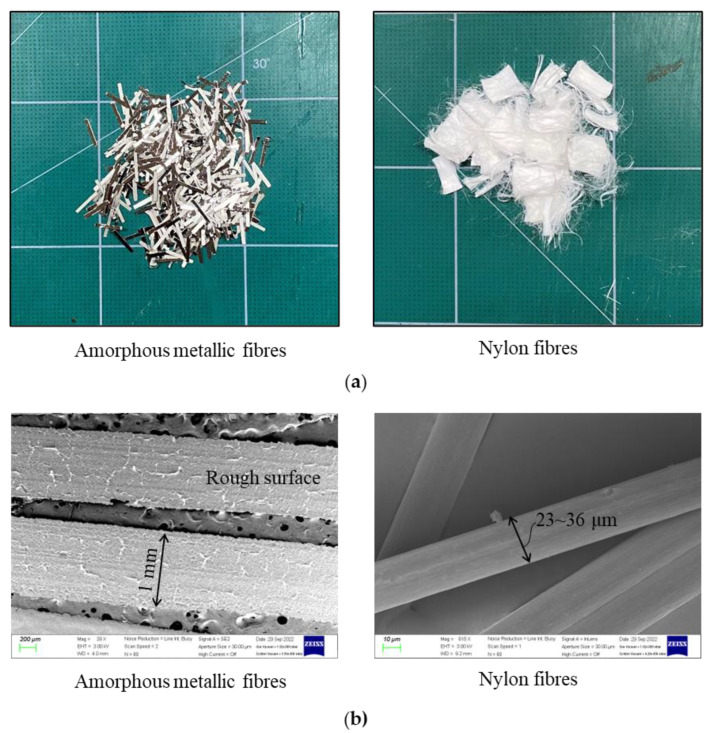
Morphology and SEM images of the fibers used in this study: (**a**) morphology of the fibers; (**b**) SEM images of the fibers.

This study utilized a hybrid form of dispersed fibers made up of amorphous metallic and nylon fibers to reinforce the cementitious matrix. Figure 4 presents the morphology (Figure 4a) and SEM images (Figure 4b) of the fibers. Table 4 lists the primary physical and mechanical properties of the fibers. The AM fibers featured a ribbon shape and rough surface and exhibited a better bonding performance between the fibers and the matrix than conventional steel fibers (Choe et al. [20], Zhao et al. [21]). Nylon fiber is a kind of synthetic polymer commonly used for concrete structures, with the capabilities of shrinkage prevention, crack width control, and increasing tensile ductility. The hybridization of the fibers was expected to achieve a balance in terms of the strength, strain capacity, and crack width of the cement-based composite. Compared to other fibers, kinds such as carbon, glass, and polyvinyl alcohol (PVA) fibers, nylon fibers are more low-cost. Specifically, they can be produced from potentially recycled products with abundant local sources to meet infrastructure demands. In the present study, AM fibers with a length of 10 mm were investigated, and nylon fibers with sizes of 6 and 12 mm were investigated, considering their effect on the workability of a cement-based mixture.

### 2.2. Test Parameters

Table 5 summarizes the test parameters and test specimens in this study. For the EG/B ratio and fiber content series, the test specimens were named following the format of “EGX-NYY-AMZ”, where X represents the EG/B ratio, Y represents the volume fraction of nylon fibers (in %), and Z represents the volume fraction of AM fibers (in %). For instance, EG0.2-NY1.0-AM0.5 represents the specimen containing 1.0% nylon and 0.5% AM fibers in the cement-based matrix.

The main test parameters included: (1) EG/B ratio, (2) fiber volume content ratio, and (3) nylon fiber length in the hybrid system. For the first parameter, the effects of the EG/B ratios of 0.2 and 0.3 were investigated for specimens containing 1.0% nylon and 0.5% AM fibers (EG0.2-NY1.0-AM0.5 and EG0.3-NY1.0-AM0.5), and for specimens containing 0.5% nylon and 1.0% AM fibers (EG0.2-NY0.5-AM1.0 and EG0.3-NY0.5-AM1.0). For the second parameter, the effect of the fiber volume content ratio was investigated. A base cement-based matrix with EG/B = 0.3 was used in this test series. Specifically, RE-EG0.3 was the specimen without fibers; for specimens EG0.3-NY1.0-AM0.5, EG0.3-NY0.75-AM0.75, and EG0.3-NY0.5-AM1.0, the volume fraction of nylon fibers and AM fibers varied from 0.5% to 1.0%, with the total volume fraction of the hybrid system kept at a constant of 1.5%. For the third parameter, the effect of the nylon fiber length was investigated. Specifically, specimens EG0.3-NY12-AM15 and EG0.3-NY6-AM15 utilized 12 mm- and 6 mm-nylon fibers, respectively. This series of specimens was fabricated with the EG/B ratio, the fiber volume fraction of nylon, and the AM fiber content corresponding to 0.3, 1.0%, and 0.5%, respectively.

### 2.3. Specimen Preparation and Mixing Procedure

Two types of specimens were prepared. The first type was used for the compressive test. According to ASTM C109/C109M [22], the cube specimens with dimensions of 50 mm × 50 mm × 50 mm were cast. The second type was used for the flexural test. According to ASTM C 1609 [23], beam specimens with dimensions of 100 mm × 100 mm × 400 mm were cast.

**Figure 5:**
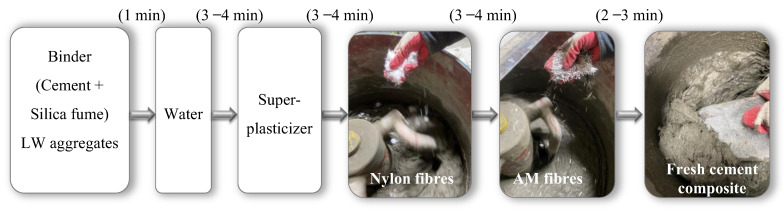
Mixing sequence of LW cement-based composites.

Figure 5 shows the mixing sequence as follows: the plain mix was prepared by first mixing the binder (cement and microsilica) and the EG particles in a pan mixer for about 1 min to achieve a uniform distribution. Water was then added and mixed for 3–4 min. Afterward, the super-plasticizer was gradually added and mixed for about 3–4 min to achieve the expected flowability. The nylon fibers were then added to the fresh mixture and mixed for an extra 3–4 min, followed by an additional 2–3 min mixing after adding the AM fibers. After mixing, the fresh mixture was poured into molds and covered by plastic layers to prevent premature water evaporation and shrinkage. The specimens were demolded after 24 h and then dry-cured for 28 days in laboratory conditions before performing tests.

### 2.4. Experimental Setup and Measuring Devices

**Figure 6:**
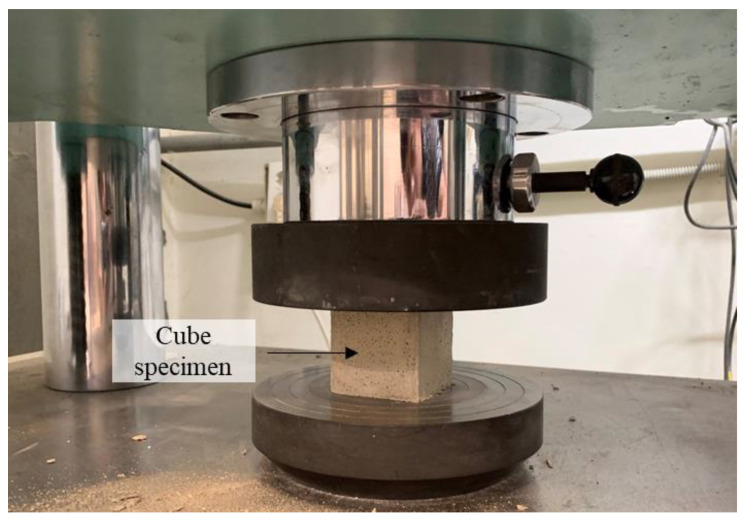
Compressive test setup.

The compressive performance of the test specimen was determined based on ASTM C109/C109 M [22]. Figure 6 presents the compressive test setup. Uniaxial compressive testing was carried out with a loading rate of 78 kN/min.

**Figure 7:**
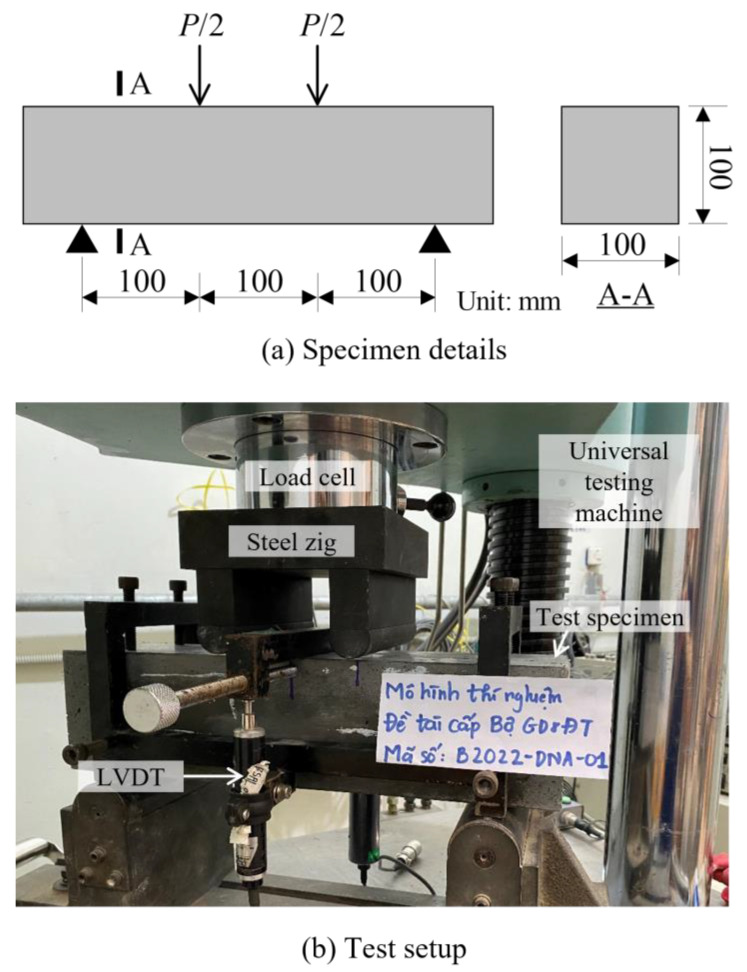
Flexural test setup.

Figure 7 presents the flexural test setup. A four-point flexural test setup was carried out in accordance with ASTM C 1609 [23], with a loading velocity of 0.05 mm/min using the displacement control method. Two linear variable differential transformers (LVDTs) were mounted on the specimens at two opposite sides to measure their deflection at the mid-span. A load cell was used to measure the loading data during the flexural tests. The flexural loading was generated through a universal testing machine (UTM) of a 1000 kN capacity. For the compressive and flexural tests, three replicas were carried out for each specimen.

## 3. Experimental Results and Discussion

### 3.1. Compressive Strength

**Figure 8:**
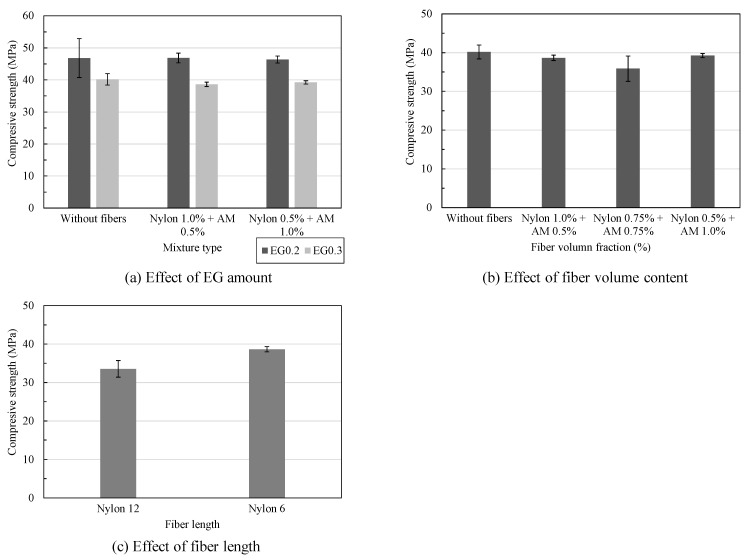
Effects of different parameters on compressive strength of lightweight cement-based composites: (**a**) effect of EG amount; (**b**) effect of fiber volume content; (**c**) effect of fiber length.

Figure 8 shows the influence of the different parameters on the compressive strength of the lightweight cement-based composites. Figure 8a indicates that increasing the EG/B ratio from 0.2 to 0.3 led to a reduction in the compressive strength, which could be attributed to the increase in the hollow degree of the matrix; however, the reduction degree was insignificant. Specifically, by increasing the EG/B ratio from 0.2 to 0.3, the specimens without fibers showed a reduction of 14.2%, the specimens containing 1.0% nylon fiber and 0.5% AM fiber showed a decrease of 17.5%, and the specimens containing 0.5% nylon fiber and 1.0% AM fiber showed a reduction of 15.3%. The densities of the cement-based matrix corresponding to the EG/B ratios of 0.2 and 0.3% were measured as 1630 and 1494 kg/m^3^, respectively. It should be noted that the densities of the LW cement-based matrices were evaluated by the water displacement method [24] after 28 days.

Figure 8b shows the effect of the fiber volume content of the hybrid type on the compressive strength of the cement-based composites. In general, the variation in the nylon and AM fiber contents in the hybrid fiber type with a total volume dosage of 1.5% insignificantly affected the compressive strength of the composite material. Compared to the control specimen without adding fibers, the cement-based mixtures containing 1.0% nylon fiber combined with 0.5% AM fiber and 0.5% nylon fiber combined with 1.0% AM fiber showed differences of only 3.80% and 2.30%, respectively, in the compressive strength. Meanwhile, the cement-based mixtures containing the same nylon and AM fiber dosage of 0.75% showed a reduction of 10.70%.

Figure 8c shows the effect of the nylon fiber length on the compressive strength of cement-based composites. The compressive strength of the mixture using 6 mm nylon fibers was measured as 38.60 MPa, which was mostly the same as the control type without fibers (40.20 MPa). The test results indicate that the compressive strength of the mixture using nylon fibers with a longer length of 12 mm led to a reduction of approximately 13% compared to that of the 6 mm nylon fibers. This could be attributed to the fact that using nylon fibers with a longer fiber length negatively affects the fiber dispersion, as they are clustered together and overlapped, leading to increases in the voids and porosity inside the matrix [25].

### 3.2. Flexural Performance

Based on the test results, the flexural properties of the materials were evaluated in accordance with ASTM C 1609 [23]. Table 6 summarizes the primary outcomes, including the flexural strength (*f_p_*), deflection peak load (*δ_p_*), and flexural toughness. The flexural strength, *f_p_*, of the test specimens was evaluated as per ASTM C 1609 [23] by the following formula:

(1) $$f_{p}=\frac{P_{p}L_{c}}{bd_{c}^{2}}$$

where *P_p_* is the peak load, *L_c_* is the distance between two supports, *b* is the width of the specimen, and *d_c_* is the height of the specimen.

**Figure 9:**
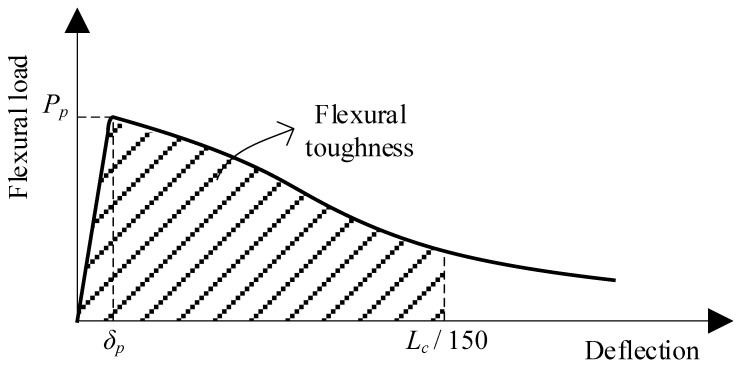
Evaluation of flexural properties in accordance with ASTM C 1609 [23].

Furthermore, the ductility of the lightweight cement-based composites represented by their flexural toughness was computed as the area under the load–deflection curves until a specific deflection value of *L_c_*/150, as shown in Figure 9. It should be noted that the toughness value of the specimen without adding fibers (RE-EG0.3) was calculated as the area under the load–deflection curves until the peak load point owing to its brittleness.

#### 3.2.1. Effect of EG/B Ratios

**Figure 10:**
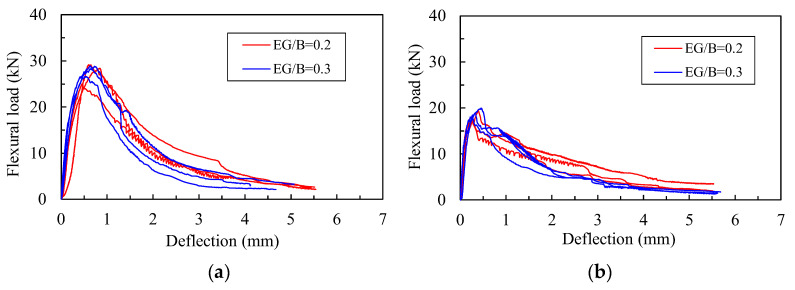
Effects of EG/B ratios on flexural load–deflection curves: (**a**) nylon 0.5% + AM 1.0%; (**b**) nylon 1.0% + AM 0.5%.

Figure 10 shows the effect of the EG/B ratios on the flexural load–deflection curves for the specimens containing 0.5% nylon fiber + 1.0% AM fiber (Figure 10a) and the specimens containing 1.0% nylon fiber + 0.5% AM fiber (Figure 10b). The results show that the amount of EG particles existing in the composites generally affected the flexural behavior of the lightweight cement-based composites less in terms of their initial stiffness, strength, and ductility.

**Figure 11:**
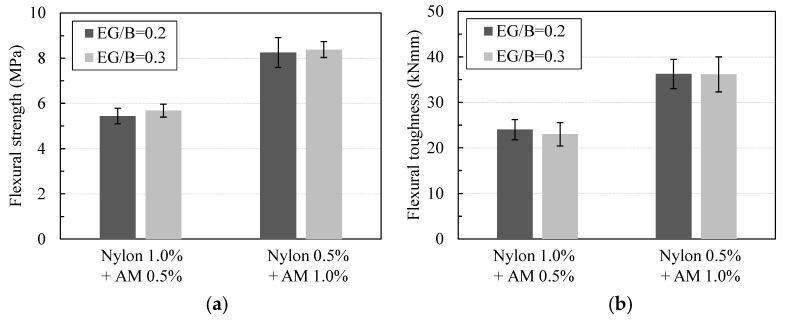
Effects of EG/B ratios on flexural properties: (**a**) on flexural strength; (**b**) on flexural toughness.

The effect of the EG/B ratio on the flexural parameters is presented in Figure 11. In Figure 11a, when increasing the EG/B ratio from 0.2 to 0.3, for the composites containing 1.0% nylon fiber incorporated with 0.5% AM fiber and 0.5% nylon fiber combined with 1.0% AM fiber, the differences in the flexural strength obtained were 4.4% and 1.5%, respectively. Meanwhile, in Figure 11b, the values regarding the differences in the flexural toughness were 4.2% and 0.2%, respectively.

#### 3.2.2. Effect of Fiber Volume Fraction Ratio

**Figure 12:**
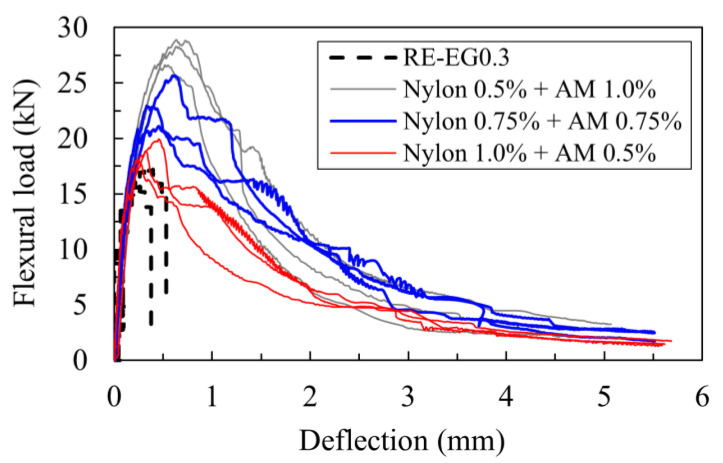
Effect of fiber volume fractions on flexural load–deflection curves.

The results in Figure 12 show the effect of the fiber volume fraction ratio on the flexural load–deflection curves. The results show that the inclusion of the hybrid fibers significantly improved the flexural behavior of the lightweight cement-based composites compared to the plain type without fibers. In addition, from the figure, the contribution of the AM and nylon fiber content ratio within a total volume fraction of the hybrid type of 1.5% was evaluated. Generally speaking, the flexural strength and deflection capacity at the peak load proportionally increased with an increasing AM fiber content in the hybrid system.

**Figure 13:**
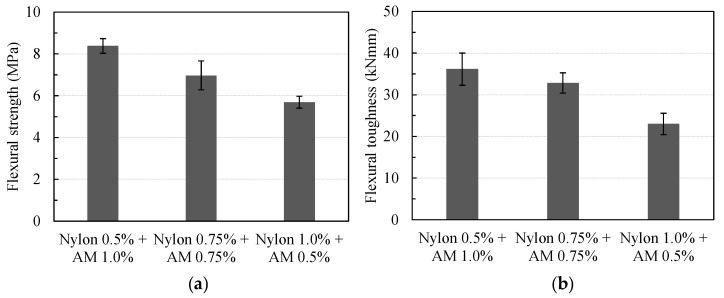
Effects of fiber volume fractions on flexural parameters: (**a**) on flexural strength; (**b**) on flexural toughness.

To be more specific, as indicated in Figure 13a, the mean flexural strength values of the specimens EG0.3-NY1.0-AM0.5, EG0.3-NY0.75-AM0.75, and EG0.3-NY0.5-AM1.0 were computed as 5.69, 6.97, and 8.38 (MPa), respectively, with COVs of 0.112, 0.074, and 0.107, respectively. Such values were approximately 14.40%, 40.30%, and 68.60% greater than that of RE-EG0.3. Additionally, increasing the AM volume content from 0.5% to 1.0% in a total fraction of 1.5% of hybrid fibers increased the mean flexural strength from 22.60% to 47.40%.

Furthermore, the inclusion of increasing the content of AM fibers within the matrix resulted in greater residual stress and flexural toughness as well. As shown in Figure 13b, the mean flexural toughness of specimens EG0.3-NY0.75-AM0.75 and EG0.3-NY0.5-AM1.0 were computed as 32.86 and 36.18 (kNmm), respectively, which were approximately 42.80%, and 57.20% greater than that of EG0.3-NY1.0-AM0.5. Compared to the specimen without fibers, RE-EG0.3, the flexural toughness of EG0.3-NY1.0-AM0.5, EG0.3-NY0.75-AM0.75, and EG0.3-NY0.5-AM1.0 improved by approximately 361.9%, 559.5%, and 626.1%, respectively.

#### 3.2.3. Effect of Nylon Fiber Length

**Figure 14:**
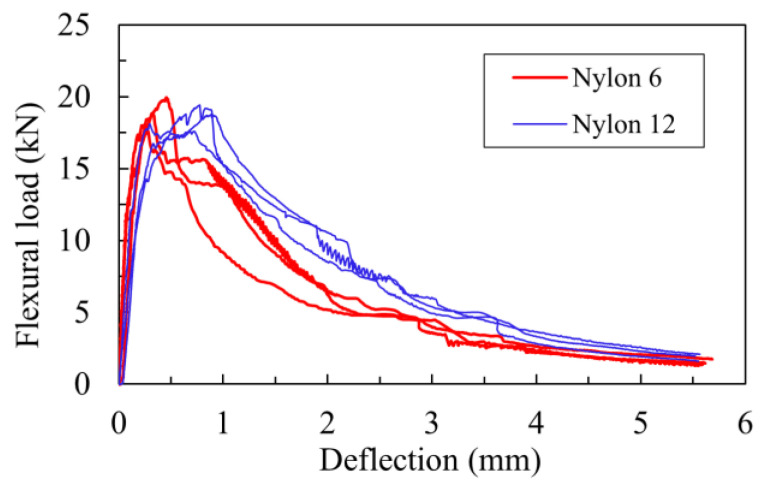
Effect of nylon fiber length on flexural load–deflection curves.

Figure 14 shows the effect of the length of nylon fibers on the flexural load–deflection curves of the lightweight cement-based composites. The results show that the nylon fiber length exhibited negligible effects on the flexural strength but significantly affected the deformation capacity at the peak load and the residual strength in the post-peak stage.

**Figure 15:**
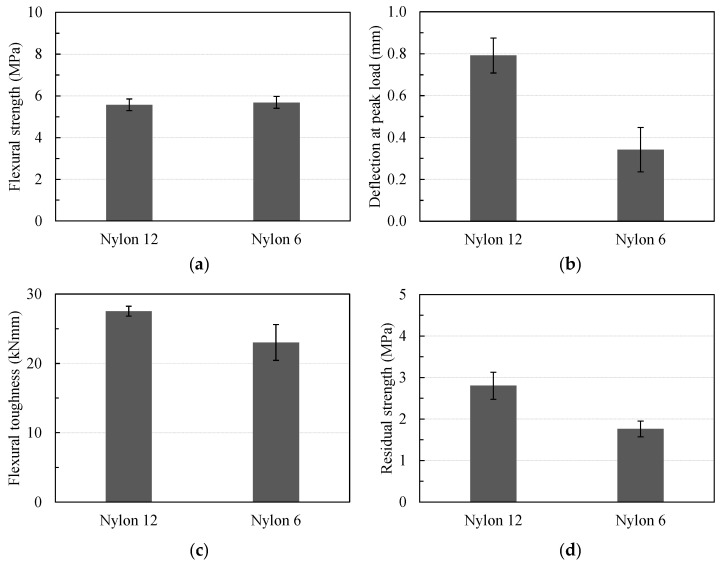
Effects of nylon fiber length on flexural parameters: (**a**) on flexural strength; (**b**) on deflection; (**c**) on flexural toughness; (**d**) on residual strength.

The effect of the nylon fiber length on the primary flexural parameters is presented in Figure 15. As shown in Figure 15a, the mean flexural strengths of specimens EG0.3-NY12-AM10 and EG0.3-NY6-AM10, using 6 mm- and 12 mm-nylon fibers, respectively, were mostly the same (5.58 and 5.69 MPa, respectively). Nevertheless, the deflection capacity at the peak load of EG0.3-NY12-AM10 was observed to be approximately two times larger than that of EG0.3-NY6-AM10 (Figure 15b).

Regarding the flexural behavior in the post-peak stage, Figure 15d shows the comparative results in terms of the residual strength of the test specimens using different nylon fiber lengths. The residual strength was obtained from the flexural stress–deflection curves after the peak load corresponding to a specific deflection value of *L_c_*/150. The results indicate that the residual strength of specimen EG0.3-NY12-AM10 using a nylon fiber length of 12 mm was 2.80 MPa, approximately 1.6 times greater than that of EG0.3-NY16-AM10 using a nylon fiber length of 6 mm. Consequently, the flexural toughness of EG0.3-NY12-AM10 was roughly 1.2 times greater than that of EG0.3-NY16-AM10, as presented in Figure 15c.

Generally speaking, the utilization of longer lengths of nylon fibers (12 mm) in the hybrid system could enhance the flexural characteristics in the post-peak stage, such as the residual resistance and flexural toughness, when compared to the shorter ones (6 mm). The results emphasize the role of longer-length fibers in enhancing the tensile stress transfer mechanism through macro-crack development after attaining the peak load to restrain the crack opening (Yu et al. [26], Park et al. [27]). Nonetheless, adverse effects regarding fiber dispersion and workability, which affect the compressive strength, should be considered to accomplish a composite with reasonable mechanical characteristics.

### 3.3. Fracture Characteristics

**Figure 16:**
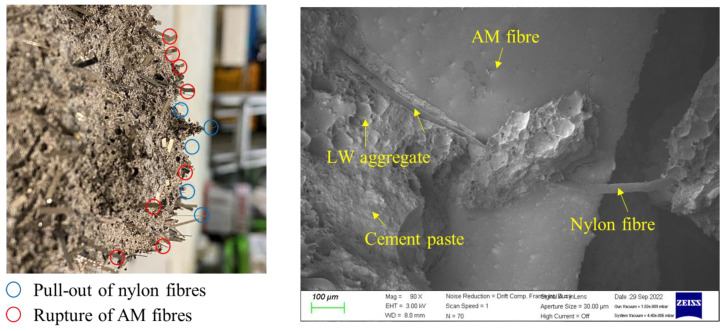
Morphology and SEM image of fracture surfaces of test specimens.

The fracture surfaces of the specimens after the flexural tests are shown in Figure 16. After the peak load, it was observed that the AM fibers were fractured, whereas the nylon fibers were pulled out. The results reveal that the AM fibers showed greater bonding strength inside the lightweight matrix than their tensile strength, leading to brittle behavior. Compared to the AM fibers, the nylon fibers showed lower bonding strength than their tensile strength and high elongation, leading to the pull-out behavior. Therefore, from a structural point of view, the high bonding strength of the AM fibers is expected to be beneficial in the flexural behavior of the composites in the pre-peak stage. Meanwhile, the pull-out characteristic of the nylon fibers is expected to be beneficial in the post-peak stage and contribute to residual stress resistance.

## 4. Conclusions

The present study developed a new type of lightweight and high-performance cement-based composite for application in thin components of buildings. For fabricating the composites, EG particles from industrial refractory materials and a hybrid fiber type made up of short AM and nylon fibers were utilized. The compressive and flexural properties of the developed cement-based composites were experimentally investigated. From the test results, the primary conclusions can be drawn as follows:

(1) Increasing the EG/B ratio from 0.2 to 0.3 resulted in a reduction of the compressive strength by a range of 14.2% to 15.3% due to the increase in the hollow extent of the matrix. A similar tendency was observed regarding the variation in the nylon fiber content of the hybrid fiber type, with a reduction in the compressive strength by a range of 2.3% to 10.7%. In addition, the utilization of nylon fibers with a longer length of 12 mm resulted in a compressive strength reduction of approximately 13% compared to that of the 6 mm nylon fibers.
(2) Regarding the fracture characteristics in flexure, the test results reveal that the AM fibers showed brittle behavior owing to a greater bonding strength inside the lightweight matrix than their tensile strength; meanwhile, the nylon fibers showed pull-out behavior due to a high elongation capacity and a lower bonding strength than their tensile strength.
(3) The amount of EG particles in the composites generally affected the flexural behavior of the lightweight cement-based composites less in terms of their initial stiffness, strength, and ductility. When increasing the EG/B ratio from 0.2 to 0.3, the difference in the flexural strength was in a range of 1.5% to 4.4%, and the values regarding the difference in flexural toughness were in a range from 0.2% to 4.2%.
(4) Increasing the AM fiber content from 0.5% to 1.0% in the hybrid system proportionally improved the mean flexural strength from 22.60% to 47.40%, as well as the deflection capacity at the peak load. In addition, in the post-peak stage, increasing the content of AM fibers within the matrix resulted in better ductility, represented by the improvement in the residual stress and flexural toughness. Compared to the specimen containing an AM volume fraction of 0.5%, the mean flexural toughness of those containing an AM volume fraction of 0.75% and 1.0% improved approximately by 42.80% and 57.20%, respectively. Such values regarding the improvement of the residual strength at a deflection of *L_c_*/150 were 78.9% and 47.7%, respectively.
(5) The nylon fiber length exhibited negligible effects on the flexural strength but significantly affected the deformation capacity at the peak load and the residual strength in the post-peak stage. The utilization of longer lengths of nylon fibers (12 mm) in the hybrid fiber type enhanced the flexural characteristics in the post-peak stage, such as the residual resistance and flexural toughness, when compared to the shorter ones (6 mm). Compared to the specimen using a 6 mm nylon fiber length, the specimen using a 12 mm nylon fiber length exhibited residual strength 1.6 times and 1.2 times higher corresponding to *L_c_*/150 and the flexural toughness, respectively.

## Figures and Tables

**Table 1:** Physical and mechanical properties of EG.

Properties	Unit	Standard	Values
Particle size	mm	ASTM C136 [13]	0.25–0.5
Fineness modulus	–	–	1.92
Bulk density	kg/m^3^	ASTM C29/C29M [14]	340
Apparent density	kg/m^3^	ASTM C128 [15]	680
Compressive strength	MPa	BS EN 13055:2016 [16]	2.6
Water absorption by mass	Mass. %	ASTM C128 [15]	28
Thermal conductivity	W/m.K	–	0.07
Color	–	–	Creamy white

**Table 2:** Mix proportions of LW cement-based composites.

Mix IDs	Mix Proportions of Matrix by Mass of Total Binder					
	W/B *	Binder		EG/B *	Sand	SP *, % by Mass of Binder
		Cement	Microsilica			
LCEG02	0.35	0.9	0.1	0.2	–	0.1
LCEG03	0.35	0.9	0.1	0.3	–	0.2

* W/B = water-to-binder ratio, EG/B = expanded glass-to-binder ratio, SP = superplasticizer.

**Table 3:** Chemical composition, clinker minerals, and physical properties of Portland cement and silica fume.

Chemical Composition	Portland Cement	Microsilica
SiO_2_, %	20.2	91.2
Al_2_O_3_, %	58.0	1.3
Fe_2_O_3_, %	3.0	0.8
CaO, %	63.3	0.7
MgO, %	3.4	0.3
SO_3_, %	2.1	-
Clinker Minerals		
C_3_S, %	54.9	-
C_2_S, %	16.6	-
C_3_A, %	10.3	-
C_4_AF, %	9.1	-
Physical properties		
Specific gravity	3.2	2.2
Fineness, m^2^/kg	312	20.47

**Table 4:** Properties of nylon and AM fibers.

Fiber Types	Length (mm)	Diameter (µm)	Width (mm)	Thickness (µm)	Density (kg/m^3^)	Elastic Modulus (GPa)
Amorphous metallic	10	-	1.0	24	7200	140
Nylon	6, 12	26	-	-	1160	2.7

**Table 5:** Details of test specimens.

Test Parameters	Specimens	EG/B Ratio	Fiber Volume Fraction (%)		Fiber Length (mm)	
			*V_f,ny_*	*V_f,AM_*	*L_f,ny_*	*L_f,AM_*
EG/B ratio	EG0.2-NY1.0-AM0.5	0.2	1.00	0.50	6	10
	EG0.2-NY0.5-AM1.0	0.2	0.50	1.00	6	10
	EG0.3-NY1.0-AM0.5 ^(1)^	0.3	1.00	0.50	6	10
	EG0.3-NY0.5-AM1.0 ^(2)^	0.3	0.50	1.00	6	10
Fiber volume content ratio	RE-EG0.3	0.3	-	-	-	-
	EG0.3-NY1.0-AM0.5 ^(1)^	0.3	1.00	0.50	6	10
	EG0.3-NY0.75-AM0.75	0.3	0.75	0.75	6	10
	EG0.3-NY0.5-AM1.0 ^(2)^	0.3	0.50	1.00	6	10
Fiber length (nylon)	EG0.3-NY12-AM10	0.3	1.00	0.50	12	10
	EG0.3-NY6-AM10 ^(1)^	0.3	1.00	0.50	6	10

^(1)^ The same specimens; ^(2)^ the same specimens.

**Table 6:** Summary of flexural test results.

Test Specimens	Flexural Characteristics							
	*f_p_* (MPa)		*δ_p_* (mm)		Toughness (KNmm)		Residual Strength at *L_c_*/150 (MPa)	
	Mean	COV	Mean	COV	Mean	COV	Mean	COV
EG0.2-NY1.0-AM0.5	5.44	0.063	0.28	0.357	24.03	0.092	2.52	0.190
EG0.2-NY0.5-AM1.0	8.26	0.080	0.63	0.311	36.26	0.089	3.46	0.180
EG0.3-NY1.0-AM0.5 ^(1)^	5.69	0.050	0.34	0.310	23.01	0.112	1.76	0.107
EG0.3-NY0.5-AM1.0 ^(2)^	8.38	0.042	0.59	0.108	36.18	0.107	2.60	0.284
RE-EG0.3	4.97	0.054	0.33	0.293	4.98	0.204	-	-
EG0.3-NY1.0-AM0.5 ^(1)^	5.69	0.050	0.34	0.310	23.01	0.112	1.76	0.107
EG0.3-NY0.75-AM0.75	6.97	0.099	0.46	0.288	32.86	0.074	3.15	0.010
EG0.3-NY0.5-AM1.0 ^(2)^	8.38	0.042	0.59	0.108	36.18	0.107	2.60	0.284
EG0.3-NY12-AM10	5.58	0.050	0.79	0.105	27.53	0.025	2.80	0.117
EG0.3-NY6-AM10 ^(1)^	5.69	0.050	0.34	0.310	23.01	0.112	1.76	0.107

^(1)^ The same specimens; ^(2)^ the same specimens.

## Data Availability

The data presented in this study are available upon request from the corresponding author.
